# Association of blood viscosity and device-free days among hospitalized patients with COVID-19

**DOI:** 10.1186/s40560-023-00665-4

**Published:** 2023-05-02

**Authors:** Ori Waksman, Daein Choi, Phyu Mar, Qinzhong Chen, Daniel J. Cho, HyoungSup Kim, Robin L. Smith, Sascha N. Goonewardena, Robert S. Rosenson

**Affiliations:** 1 Metabolism and Lipids Unit, Cardiovascular Institute, Marie-Josee and Henry R Kravis Center for Cardiovascular Health, Mount Sinai Heart, Icahn School of Medicine at Mount Sinai, The Mount Sinai Medical Center, One Gustave L. Levy Place, Box 1030, New York, NY 10029 USA; 2 Department of Medicine, Mount Sinai Beth Israel, Icahn School of Medicine at Mount Sinai, New York, NY USA; 3 Rheovector LLC, King of Prussia, PA USA; 4 CURA Foundation, New York, NY USA; 5 Division of Cardiovascular Medicine, Department of Internal Medicine, University of Michigan, Ann Arbor, MI USA

**Keywords:** Blood viscosity, Coronavirus-19, Critical care medicine, Hypoxic respiratory failure

## Abstract

**Background:**

Increased estimated whole blood viscosity (eWBV) predicts higher mortality in patients hospitalized for coronavirus disease 2019 (COVID-19). This study assesses whether eWBV is an early predictor of non-fatal outcomes among patients hospitalized for acute COVID-19 infection.

**Methods:**

This retrospective cohort study included 9278 hospitalized COVID-19 patients diagnosed within 48 h of admission between February 27, 2020 to November 20, 2021 within the Mount Sinai Health System in New York City. Patients with missing values for major covariates, discharge information, and those who failed to meet the criteria for the non-Newtonian blood model were excluded. 5621 participants were included in the main analysis. Additional analyses were performed separately for 4352 participants who had measurements of white blood cell count, C-reactive protein and D-dimer. Participants were divided into quartiles based on estimated high-shear blood viscosity (eHSBV) and estimated low-shear blood viscosity (eLSBV). Blood viscosity was calculated using the Walburn–Schneck model. The primary outcome was evaluated as an ordinal scale indicating the number of days free of respiratory organ support through day 21, and those who died in-hospital were assigned a value of -1. Multivariate cumulative logistic regression was conducted to evaluate the association between quartiles of eWBV and events.

**Results:**

Among 5621 participants, 3459 (61.5%) were male with mean age of 63.2 (SD 17.1) years. The linear modeling yielded an adjusted odds ratio (aOR) of 0.68 (95% CI 0.59–0.79, *p* value < 0.001) per 1 centipoise increase in eHSBV.

**Conclusions:**

Among hospitalized patients with COVID-19, elevated eHSBV and eLSBV at presentation were associated with an increased need for respiratory organ support at 21 days. These findings are highly relevant, as they demonstrate the utility of eWBV in identifying hospitalized patients with acute COVID-19 infection at increased risk for non-fatal outcomes in early stages of the disease.

## Introduction

Despite major developments in anti-viral therapies and widespread vaccination campaigns, emerging variants of coronavirus disease 2019 (COVID-19) continue to pose public health challenges [1]. While the disease course for most individuals who contract COVID-19 is limited to mild respiratory symptoms, some patients progress to severe respiratory dysfunction, multi-organ failure and death [2, 3]. The early identification of patients at high risk for clinical deterioration is paramount, as anti-viral trials for COVID-19 have demonstrated associations between early clinical intervention and the decreased risk of disease progression.

To date, models attempting to risk stratify patients for the development of severe respiratory and systemic disease have chiefly utilized hemostatic and hepatically derived inflammatory biomarkers as surrogate measures of disease severity [4]. However, contemporary studies of COVID-19 etiopathology, suggest that the hyperinflammatory response involves the release of a complex network of inflammatory, immune and coagulation mediators, which may not be appropriately reflected in these traditional laboratory measures [5–7].

Emerging studies have supported the use of whole blood viscosity (WBV) as a prognostic measure of COVID-19 disease severity [8, 9]. WBV is a validated measure of rheology and is chiefly determined by hematocrit, plasma viscosity, and RBC deformability [10]. It is proposed that the derangements of inflammatory mediators in the setting of COVID-19, contribute to altered blood rheology and thus WBV may be reflective of the overall hyperinflammatory and hypercoagulable state induced by COVID-19.

In a recently published investigation, estimated whole blood viscosity (eWBV) was shown to identify patients with a higher mortality risk after hospitalization for acute COVID-19 infection. The associations between mortality, elevated high-shear eWBV (eHSBV) and low-shear eWBV (eLSBV) were significant after adjustment for age, sex, cardiometabolic comorbidities, or in-hospital pharmacotherapy. That study revealed that one centipoise (cP) increase in eHSBV and eLSBV was associated with 36% and 7% increased risk of in-hospital mortality, respectively (*p* < 0.0001) [8]. Notably, when compared to common inflammatory biomarkers routinely used by clinicians for risk stratification [white blood cell count (WBC), C-reactive protein (CRP) and D-dimer], eWBV was found to be a superior predictor of mortality.

As the endpoint of mortality does not reflect non-fatal COVID-19-related clinical outcomes, we aimed to determine whether WBV could predict the need for oxygen dependence throughout a given hospitalization. Utilizing the validated outcome measure of respiratory organ support-free days up to 21 days [11], we hypothesize that WBV will be associated with an increased need for respiratory organ support at 21 days and may be a better predictor of non-fatal outcome among hospitalized patients with COVID-19, when compared to commonly used laboratory biomarkers and recognized demographic and cardiometabolic risk factors.

## Methods

### Data collection

The study population is derived from the Mount Sinai Health System (MSHS) COVID-19 database, which was collected from the electronic health records of six hospitals within MSHS: Mount Sinai Beth Israel, Mount Sinai Brooklyn, Mount Sinai Hospital, Mount Sinai Morningside, Mount Sinai Queens and Mount Sinai West. The collected data include demographic information (age, sex and ethnicity) obtained at the time of admission, past medical history, biometric and laboratory data during the hospital stay and dispensed medication.

### Patient population

**Fig. 1:**
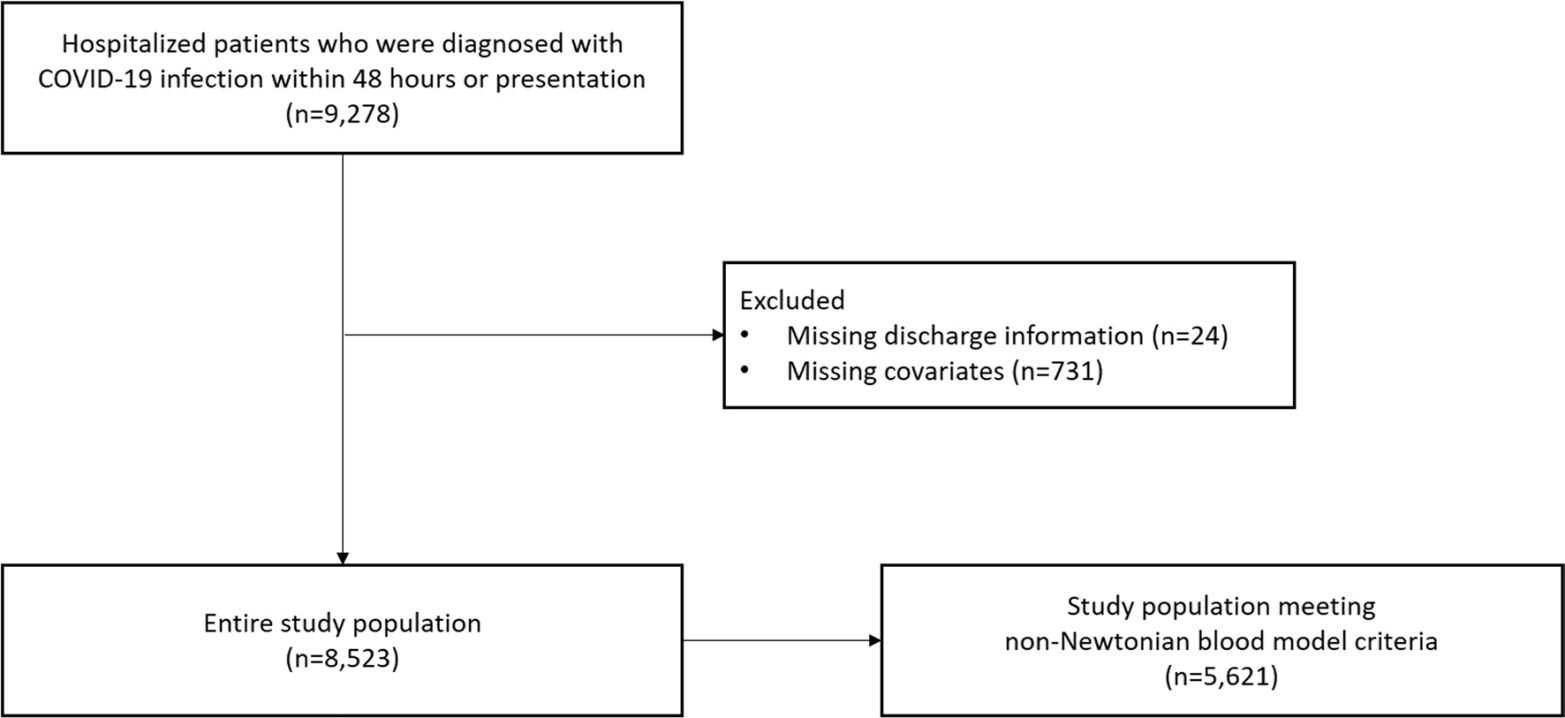
Flow diagram of the study population

A total of 9278 hospitalized patients with COVID-19 who were diagnosed within 48 h from admission between February 27, 2020 to November 20, 2021 were included. The diagnosis of COVID-19 infection was defined as a positive reverse transcriptase-polymerase chain reaction assay. 24 individuals with missing data of discharge information and 731 participants with missing values for major covariates (hemoglobin, total protein and albumin level within 24 h of presentation) were excluded. Lastly, we excluded 2902 patients who do not meet the criteria for the non-Newtonian blood model [12]. A total of 5621 study participants were included in the main analysis (Fig. 1). The validated non-Newtonian blood model includes hematocrit dependence, which refers to a hematocrit range between 37 to 55% [12]. The Walburn–Schneck model was used for calculations of eHSBV and eLSBV among hospitalized patients with acute COVID-19 according to these formulas.

$$\mathrm{eLSBV}= 0.00797{e}^{0.0608 [\mathrm{Hematocrit}]} [{e}^{14.585(\mathrm{Total \,Protein \, Minus \, Albumin }/ [\mathrm{Hematocrit}^2]}] {(5)}^{-0.00499(\mathrm{Hematocrit})} ,$$

$$\mathrm{eHSBV}= 0.00797{e}^{0.0608 [\mathrm{Hematocrit}]} \left[{e}^{14.585(\mathrm{Total \, Protein \, Minus \, Albumin }/ [\mathrm{Hematocrit}^2]}\right]{(300)}^{-0.00499\left(\mathrm{Hematocrit}\right).10-12}$$

This Walburn–Schneck model was validated in a separate cohort of COVID-19 patients who had direct measurements of WBV [9].

Study participants were divided into quartiles based on eHSBV and eLSBV. The primary outcome was evaluated as an ordinal scale indicating the number of days free of respiratory organ support through day 21, and those who died in-hospital were assigned a value of − 1. Respiratory organ support was defined by the need for high-flow nasal cannula, invasive or noninvasive ventilation, or extracorporeal life support. This measure of outcome was used as a measure of prognosis and validated in previous studies in patients with COVID-19 [11].

### Statistical analysis

Continuous variables were reported as mean and standard deviation, and categorical variables were reported as counts and percentages. Analysis of variance tests were performed for continuous variables and Chi-square tests were conducted for categorical variables to assess the distribution difference between four quartiles. Multivariate cumulative logistic regression was conducted to evaluate the association between eWBV and the primary outcome. Considered covariates included age, sex, hospital site, race, past medical history of hypertension, diabetes mellitus, chronic kidney disease, and coronary artery disease, in-hospital statin therapy, in-hospital anticoagulation therapy, date of admission, and measure of initial oxygen support. Additional analysis was conducted among 4352 participants with measurement of inflammatory markers (white blood cell count (WBC), C-reactive protein (CRP) and D-dimer). WBC, CRP, and D-dimer were specifically collected as these are among the most routinely available and widely used inflammatory biomarkers. These biomarkers additionally served as a baseline to compare the prognostic value of eWBV to the current standard of care in evaluating respiratory organ support-free days. Supplementary analysis among 495 participants with measurement of arterial blood gas at the time of presentation was performed with PaO_2_/FiO_2_ ratio as an additional covariate instead of initial measure of oxygen support.

Stratified analyses were performed according to the subgroups of age, sex, race, comorbidities, admission date, in-hospital pharmacotherapy, initial oxygen support and Intensive Care Unit (ICU) admission.

All data collection and statistical analyses were conducted with SAS Enterprise Guideline 8.3 (SAS Institute, Cary, NC, USA). A two-sided *p*-value of < 0.05 was used to define the statistical significance. This study was approved by the Institutional Review Board of Icahn School of Medicine at Mount Sinai (IRB number: 20-03558). Patient consent was waived as the database is anonymized according to strict confidentiality guidelines prior to distribution.

## Results

Descriptive characteristics of the study population are shown in Table 1. Participants with higher eHSBV were more likely be male, under 65 years, of Black or Hispanic ethnicity, diagnosed with diabetes mellitus, and required higher levels of oxygen support early in the course of hospitalization. Additionally, initial levels of inflammatory markers tended to be higher and PaO_2_/FiO_2_ ratio was lower among those with higher eHSBV. There were no statistically significant differences in in-hospital pharmacotherapy or comorbidities other than diabetes between the four study groups.

**Table 1:** Descriptive characteristics of study population

	High-shear BV quartile 1 (Lowest)	High-shear BV quartile 2	High-shear BV quartile 3	High-shear BV quartile 4 (Highest)	*p*-value
Number of participants, N	1405	1406	1405	1405	
Blood viscosity range (cP)	3.01–4.00	4.00–4.24	4.24–4.53	4.53–9.86	
*Sex, N (%)*					< 0.001
Men	663 (46.2)	819 (58.3)	929 (33.9)	1,048 (74.6)	
Women	742 (52.8)	587 (41.8)	476 (66.1)	357 (25.4)	
Age, mean (SD)	64.9 (17.4)	63.2 (17.0)	62.8 (16.5)	62.0 (17.3)	< 0.001
*Race, N (%)*					< 0.001
White	536 (38.2)	359 (24.9)	314 (22.4)	279 (19.9)	
Black	242 (17.2)	312 (22.2)	308 (21.9)	333 (23.7)	
Asian	92 (6.6)	95 (6.8)	81 (5.8)	69 (4.9)	
Hispanic	311 (22.1)	387 (27.5)	429 (30.5)	443 (31.5)	
Others	224 (15.9)	262 (18.6)	273 (19.4)	281 (20.0)	
*Comorbidity, N (%)*					
HTN	458 (32.6)	472 (33.6)	484 (33.7)	437 (31.1)	0.424
DM	239 (17.0)	292 (20.8)	285 (20.3)	296 (21.1)	0.025
CKD	76 (5.4)	79 (5.6)	92 (6.6)	85 (6.1)	0.588
CAD	172 (12.2)	175 (12.5)	154 (11.0)	170 (12.1)	0.619
*Admission date*					< 0.001
3/1/2020–8/31/2020	622 (44.3)	689 (49.0)	742 (52.8)	771 (54.9)	
9/1/2020–2/28/2021	568 (40.4)	523 (37.2)	479 (34.1)	429 (30.5)	
3/1/2021–11/20/2021	215 (15.3)	194 (13.8)	184 (13.1)	205 (14.6)	
*Initial oxygen support device*					< 0.001
Room air	487 (34.7)	401 (28.5)	345 (24.6)	322 (22.9)	
NC or NRB or HFNC	827 (58.9)	890 (63.3)	888 (63.2)	888 (63.2)	
BiPAP or CPAP	59 (4.2)	84 (6.0)	112 (8.0)	141 (10.0)	
Intubated	32 (2.3)	31 (2.2)	60 (4.3)	54 (3.8)	
*Anticoagulation*					0.072
No anticoagulation	93 (6.6)	59 (4.2)	81 (5.8)	76 (5.4)	
Prophylactic dose	578 (41.1)	601 (42.8)	613 (42.6)	569 (40.5)	
Therapeutic dose	734 (52.2)	746 (53.1)	711 (50.6)	760 (54.1)	
Participants with lab data	1006	1094	1116	1136	
WBC, × 10^3^/uL	7.5 (4.1)	8.0 (4.4)	8.5 (3.9)	9.4 (5.4)	< 0.001
CRP, mg/L	94.0 (84.5)	113.1 (87.1)	124.1 (93.6)	126.3 (96.9)	< 0.001
D-dimer, ug/mL	1.9 (3.1)	2.5 (4.0)	2.6 (4.2)	4.2 (6.2)	< 0.001
Participants with initial arterial blood gas	102	97	129	167	
PaO_2_/FiO_2_ ratio ≥ 300	25 (24.5)	14 (14.4)	13 (10.1)	23 (13.8)	0.056
200 ≤ PaO_2_/FiO_2_ ratio < 300	12 (11.8)	8 (8.3)	23 (17.8)	17 (10.2)	
100 ≤ PaO_2_/FiO_2_ ratio < 200	36 (35.3)	35 (36.1)	43 (33.3)	62 (37.1)	
PaO_2_/FiO_2_ ratio < 100	29 (28.4)	40 (41.2)	50 (38.8)	65 (38.9)	

N, number of participants; SD, standard deviation; HTN, hypertension; DM, diabetes mellitus; CKD, chronic kidney disease; CAD, coronary artery disease; NC, nasal cannula; NRB, non-rebreather mask; HFNC, high-flow nasal cannula; BiPAP, bilevel positive airway pressure; CPAP, continuous positive airway pressure; SBP, systolic blood pressure; WBC white blood cell count; CRP, C-reactive protein

Table 2 shows the association between eHSBV and respiratory organ support-free days in hospitalized patients with COVID-19. The eHSBV range was categorized into quartiles, with the first quartile being 3.01–4.00 cP, the second being 4.00–4.24 cP, the third being 4.24–4.53 cP, and the fourth being 4.53–9.86 cP. Compared to participants in the lowest quartile of eHSBV, individuals in the highest quartile of eHSBV had lower odds for respiratory organ support-free days, yielding an adjusted odds ratio (aOR) of 0.65 (95% confidence interval [CI]; 0.54–0.78). Similarly, participants in the second and third quartiles of eHSBV had lower odds for respiratory organ support-free days compared to those in the lowest quartile of eHSBV, with aOR of 0.83 (95% CI 0.69–0.99) and 0.81 (95% CI 0.68–0.97), respectively. On average, participants in the highest quartile of eHSBV had 14.5 respiratory organ support-free days compared to 17.0 days among those in the lowest quartile. Participants with higher eHSBV had lower odds for respiratory organ support-free days even after adjustment of inflammatory markers (aOR 0.71, 95% CI 0.58–0.88). The linear modeling yield aOR of 0.68 (95% CI 0.59–0.79, *p* value < 0.001) per 1 centipoise increase in eHSBV.

**Table 2:** Association of high-shear blood viscosity and respiratory organ support-free days up to day 21

	High-shear BV quartile 1 (Lowest)	High-shear BV quartile 2	High-shear BV quartile 3	High-shear BV quartile 4 (Highest)	*p* for trend
*N*	1405	1406	1405	1405	
Blood viscosity range (cP)	3.01–4.00	4.00–4.24	4.24–4.53	4.53–9.86	
aOR (95% CI)	1.00 (reference)	0.83 (0.69–0.99)	0.81 (0.68–0.97)	0.65 (0.54–0.78)	< 0.001
aOR^+^ (95% CI)	1.00 (reference)	0.87 (0.71–1.06)	0.89 (0.73–1.09)	0.71 (0.58–0.88)	0.003
Mean respiratory organ support-free days (standard deviation)	17.0 (7.9)	16.1 (8.6)	15.6 (8.8)	14.5 (9.4)	

Linear modeling result: aOR 0.68 (95% CI 0.59–0.79, *p* < 0.001) per 1 cP increase; aOR 0.82 (95% CI 0.76–0.88, *p* < 0.001) per IQR (interquartile range, 0.53 cP) increase

Adjusted odds ratios calculated by cumulative logistic regression after adjustments for age, sex, hospital site, race, history of HTN, DM, CKD, and CAD, in-hospital statin use, anticoagulation therapy, date of admission, and measure of initial oxygen support

BV, blood viscosity; cP, centipoise; aHR, adjusted hazard ratio; CI, confidence interval; HTN, hypertension; DM, diabetes mellitus; CKD, chronic kidney disease; CAD, coronary artery disease

^+^ Adjusted odds ratios calculated by cumulative logistic regression after adjustments for age, sex, hospital site, race, history of HTN, DM, CKD, and CAD, in-hospital statin use, anticoagulation therapy, date of admission, measure of initial oxygen support, and initial lab data (white blood cell count, CRP, and D-dimer)

The association between eLSBV and respiratory organ support-free days is depicted in Table 3. The range of eLSBV was 6.49–9.05 cP, 9.05–10.01 cP, 10.01–11.29 cP and 11.29–25.50 cP for the first, second, third, and fourth quartiles of eLSBV, respectively. Participants with the highest eLSBV were less likely to have respiratory organ support-free days compared to those with the lowest eLSBV, yielding an aOR of 0.67 (95% CI 0.56–0.80). A similar association was observed with an aOR of 0.70 (95% CI 0.57–0.86) in models that adjusted for inflammatory markers. Participants in the highest quartile of eLSBV had an average of 14.9 respiratory organ support-free days, whereas those in the lowest quartile had an average of 16.7 respiratory organ support-free days. One centipoise increase in eLSBV was associated with lower odds for respiratory organ support-free days (aOR 0.91, 95% CI 0.88–0.95, *p* value < 0.001) in the linear modeling.

**Table 3:** Association of shear blood viscosity and respiratory organ support-free days up to day 21

	Low-shear BV quartile 1 (Lowest)	Low-shear BV quartile 2	Low-shear BV quartile 3	Low-shear BV quartile 4 (Highest)	*p* for trend
*N*	1405	1406	1405	1405	
Blood viscosity range (cP)	6.49–9.05	9.05–10.01	10.01–11.29	11.29–25.50	
aOR (95% CI)	1.00 (reference)	0.84 (0.70–0.99)	0.80 (0.67–0.960)	0.67 (0.56–0.80)	< 0.001
aOR^+^ (95% CI)	1.00 (reference)	0.88 (0.72–1.07)	0.83 (0.68–1.01)	0.70 (0.57–0.86)	< 0.001
Mean respiratory organ support-free days (standard deviation)	16.7 (8.1)	16.1 (8.6)	15.6 (8.8)	14.9 (9.2)	

Linear modeling result: aOR 0.91 (95% CI 0.88–0.95, *p* < 0.001) per 1 cP increase; aOR 0.82 (95% CI 0.76–0.88, *p* < 0.001) per IQR (interquartile range, 2.24 cP) increase

Adjusted odds ratios calculated by cumulative logistic regression after adjustments for age, sex, hospital site, race, history of HTN, DM, CKD, and CAD, in-hospital statin use, anticoagulation therapy, date of admission, and measure of initial oxygen support

BV, blood viscosity; cP, centipoise; aHR, adjusted hazard ratio; CI, confidence interval; HTN, hypertension; DM, diabetes mellitus; CKD, chronic kidney disease; CAD, coronary artery disease

^+^ Adjusted odds ratios calculated by cumulative logistic regression after adjustments for age, sex, hospital site, race, history of HTN, DM, CKD, and CAD, in-hospital statin use, anticoagulation therapy, date of admission, measure of initial oxygen support, and initial lab data (white blood cell count, CRP, and D-dimer)

Table 4 shows the results of subgroup analyses. Participants with higher eHSBV were consistently associated with lower odds for respiratory organ support-free during hospitalization among multiple subgroups of age, sex, race, comorbidities, in-hospital pharmacotherapy and initial measure of oxygen support. This association was more prominent among participants with older age (aOR 0.59, 95% CI 0.47–0.75), Hispanic (aOR 0.59, 95% CI 0.41–0.85), with history of coronary artery disease (aOR 0.52, 95% CI 0.31–0.86) and intubated during hospitalization (aOR 0.36, 95% CI 0.14–0.94).

**Table 4:** Association of high-shear blood viscosity and respiratory organ support-free days up to day 21 according to subgroups

	HSBV quartile 1 (Lowest)	HSBV quartile 2	HSBV quartile 3	HSBV quartile 4 (Highest)	*p* for trend
*Age*					
≥ 65 years	1.00 (reference)	0.81 (0.65–1.02)	0.79 (0.63–0.99)	0.59 (0.47–0.75)	< 0.001
< 65 years	1.00 (reference)	0.90 (0.67–1.20)	0.94 (0.70–1.25)	0.79 (0.59–1.05)	0.119
*Sex*					
Men	1.00 (reference)	0.80 (0.63–1.01)	0.82 (0.65–1.04)	0.64 (0.52–0.82)	< 0.001
Women	1.00 (reference)	0.88 (0.67–1.15)	0.80 (0.60–1.06)	0.61 (0.45–0.83)	0.002
*Race*					
White	1.00 (reference)	0.69 (0.50–0.95)	0.66 (0.48–0.92)	0.60 (0.43–0.84)	0.002
Black	1.00 (reference)	0.94 (0.62–1.42)	0.88 (0.58–1.34)	0.73 (0.48–1.09)	0.100
Asian	1.00 (reference)	1.29 (0.63–2.67)	2.60 (1.17–5.81)	1.13 (0.52–2.44)	0.369
Hispanic	1.00 (reference)	0.84 (0.58–1.22)	0.77 (0.53–1.10)	0.59 (0.41–0.85)	0.003
Other races	1.00 (reference)	0.84 (0.55–1.28)	0.87 (0.57–1.33)	0.68 (0.45–1.04)	0.098
*HTN*					
Yes	1.00 (reference)	1.05 (0.78–1.42)	0.96 (0.72–1.29)	0.75 (0.55–1.01)	0.039
No	1.00 (reference)	0.71 (0.57–0.89)	0.74 (0.59–0.92)	0.59 (0.47–0.74)	< 0.001
*DM*					
Yes	1.00 (reference)	0.87 (0.59–1.29)	1.06 (0.71–1.58)	0.74 (0.50–1.10)	0.228
No	1.00 (reference)	0.84 (0.69–1.03)	0.76 (0.62–0.93)	0.64 (0.52–0.79)	< 0.001
*CKD*					
Yes	1.00 (reference)	0.73 (0.35–1.54)	0.99 (0.48–2.06)	0.44 (0.22–0.90)	0.792
No	1.00 (reference)	0.84 (0.70–1.01)	0.80 (0.67–0.96)	0.68 (0.57–0.82)	< 0.001
*CAD*					
Yes	1.00 (reference)	0.70 (0.42–1.16)	0.75 (0.45–1.25)	0.52 (0.31–0.86)	0.021
No	1.00 (reference)	0.84 (0.69–1.01)	0.82 (0.68–0.99)	0.67 (0.55–0.81)	< 0.001
*Comorbidities*					
Yes	1.00 (reference)	0.94 (0.73–1.21)	0.94 (0.73–1.22)	0.67 (0.52–0.87)	0.003
No	1.00 (reference)	0.75 (0.58–0.96)	0.70 (0.54–0.90)	0.62 (0.48–0.80)	0.004
*Admission date*					
03/2020–08/2020	1.00 (reference)	0.93 (0.72–1.21)	0.84 (0.65–1.09)	0.66 (0.51–0.85)	< 0.001
09/2020–02/2021	1.00 (reference)	0.77 (0.58–1.03)	0.79 (0.59–1.06)	0.56 (0.41–0.75)	< 0.001
03/2021–11/2021	1.00 (reference)	0.67 (0.41–1.10)	0.78 (0.47–1.31)	0.86 (0.52–1.42)	0.755
*Statin therapy*					
Yes	1.00 (reference)	0.78 (0.58–1.05)	0.85 (0.63–1.16)	0.74 (0.54–0.99)	0.093
No	1.00 (reference)	0.85 (0.68–1.06)	0.81 (0.65–1.01)	0.62 (0.50–0.78)	< 0.001
*Anticoagulation*					
No	1.00 (reference)	2.47 (0.50–12.3)	0.99 (0.27–3.67)	0.73 (0.19–2.84)	0.281
Prophylactic	1.00 (reference)	1.01 (0.74–1.36)	0.95 (0.70–1.29)	0.67 (0.49–0.90)	0.008
Therapeutic	1.00 (reference)	0.71 (0.57–0.89)	0.74 (0.59–0.93)	0.63 (0.50–0.79)	< 0.001
*Initial o2 support*					
None	1.00 (reference)	1.07 (0.62–1.83)	1.23 (0.68–2.22)	0.72 (0.42–1.24)	0.344
O2 support other than intubation	1.00 (reference)	0.82 (0.67–1.01)	0.77 (0.63–0.95)	0.62 (0.50–0.76)	< 0.001
Intubated	1.00 (reference)	0.26 (0.08–0.78)	0.29 (0.11–0.78)	0.36 (0.14–0.94)	0.101
*ICU admission*					
Yes	1.00 (reference)	0.53 (0.37–0.75)	0.75 (0.53–1.07)	0.66 (0.47–0.94)	0.161
No	1.00 (reference)	0.92 (0.72–1.16)	0.77 (0.61–0.98)	0.57 (0.45–0.71)	< 0.001

Adjusted odds ratios calculated by cumulative logistic regression after adjustments for age, sex, hospital site, race, history of HTN, DM, CKD, and CAD, in-hospital statin use, anticoagulation therapy, date of admission, measure of initial oxygen support, and initial lab data (white blood cell count, CRP, and D-dimer)

HSBV, high-shear blood viscosity; cP, centipoise; aHR, adjusted hazard ratio; CI, confidence interval; HTN, hypertension; DM, diabetes mellitus; CKD, chronic kidney disease; CAD, coronary artery disease

## Discussion

Among hospitalized patients with acute COVID-19 infection, increased eHSBV and eLSBV on admission were both associated with a decreased likelihood of being respiratory organ support-free at 21 days (aOR 0.65; CI 0.54–0.78 and aOR 0.67, 95% CI 0.56–0.78, respectively (*p* value < 0.001). This association was consistent after adjustment age, sex and cardiometabolic comorbidities; inflammatory biomarkers including WBC, CRP, D-dimer, WBC; and pharmacotherapy. The result of this study builds on the findings of our parallel investigation exploring the endpoint of all-cause mortality [8], and demonstrates the ability of WBV to predict non-fatal COVID-19 outcomes among hospitalized patients early in the disease course.

Historically, WBV has been clinically utilized as predictor of cardiovascular risk, with elevations of WBV carrying associations with plaque rupture, and vascular compromise in otherwise healthy individuals [10, 13, 14]. Previous investigations of WBV in the setting of COVID-19 have shown that HSBV and LSBV remains for up to 8 weeks into the convalescent stage of the illness. When measured with a scanning capillary viscometer (Hemathix™ Blood Analyzer; RheoVector, LLC, King of Prussia, PA, USA), healthy controls had a BV of 4.2 cP and 13.0 cP at 300 s^–1^ and 5 s^–1^ while patients with acute COVID infection had a BV of 5.1 cP and 16.0 cP at 300 s^–1^and 5 s^–1^, respectively [9]. Our studies are among the first large investigations to use blood viscosity to prognosticate outcome in the setting of viral illness.

Mechanistically, we hypothesize that in the setting of COVID-19 high concentrations of acute phase proteins increase plasma viscosity due to their large molecular mass, raising serum viscosity in a manner analogous to that observed with hematological malignancies with high levels of paraproteins [15, 16]. The presence of these charged inflammatory and immune mediators alter erythrocyte–erythrocyte interactions contributing to increased aggregation, decreased deformability and poor laminar flow of the erythrocyte.

As whole blood is a non-Newtonian fluid, viscosity is dependent on shear rate with high HSBV measured at 300 s^–1^ and LSBV measured at 5 s^–1^. In areas of high shear, increased blood viscosity results in mechanical trauma to the endothelium leading to plaque instability, impaired oxygen delivery and further activation of pro-inflammatory cascades [17–19]. In areas of low shear, increased blood viscosity fosters erythrocyte aggregation leading to sluggish turbulent flow, microvascular stasis, and increased thrombotic risk through mechanisms of Virchow’s triad. Given the high prevalence of arterial, venous and microvascular thrombosis observed in COVID-19, we postulate that rheological measures of WBV may be more physiologically relevant than inflammatory biomarkers [2, 3, 10, 13, 14, 17–19].

Our study found that WBV was a reliable predictor of outcome largely independent of initial disease severity. Among those presenting to the hospital without oxygen support, patients in the highest quartile of eHSBV demonstrated a decreased likelihood of no respiratory organ support at 21 days when compared to the lowest quartile of eHSBV (aOR of 0.72; CI 0.42–1.24 and aOR 1.07 CI 0.57–0.89, respectively). Similar trends were observed among those presenting with oxygen support other than intubation (aOR of 0.62; CI 0.50–0.76 and aOR 0.82 CI 0.67–1.01, respectively). This trend was not observed among patients who were intubated on admission, which may be due to low power of this cohort or overall poor prognosis from advanced disease. This finding is especially notable as it demonstrates the ability of WBV to predict disease course at time of hospital admission largely independent of disease acuity.

From a respiratory perspective, autopsy studies of patients with COVID-19 ARDS have confirmed a significantly higher prevalence of arterial and venous thrombosis, when compared to equally severe influenza infection [2]. The development of diffuse microvascular thrombosis and microangiopathy of the pulmonary vasculature contributes to the development of COVID-19 acute respiratory distress syndrome (ARDS) via ventilation/perfusion defects, including shunting and decreased space [20]. We hypothesize that increases in WBV particularly in low shear areas such the pulmonary capillaries may increase the risk of thrombosis and microangiopathy thereby contributing to ARDS. Further investigation regarding the associations of WBV and ARDS is warranted.

When comparing the aOR of the highest quartile by admission date, patients admitted between 3/2021–11/2021, reflected a better likelihood of becoming respiratory organ support free at 21 days (aOR 0.86) than those admitted between 3/2020–08/2020 and 9/2020–2/2021 (aOR 0.66 and 0.56, respectively). We postulate this may be due to increased prevalence of vaccinated patients during that time frame. Additionally, the aOR was different in individuals age over 65 years (0.59 95% CI, 0.47–0.75) versus age lower than 65 years (0.79, 95% CI 0.59–1.05), reflecting increased likelihood of poor outcome among the younger cohort with increased viscosity. The lower aOR among the younger patient cohort may signify that inflammation plays a more significant role in the absence of other comorbidities or may reflect of a tendency of a hospital protocol to selectively admit more critically ill younger individuals; however, this supposition warrants further investigation.

Our study has a few limitations to consider. First, eBV was calculated using the Walburn–Schneck model and not directly measured. When evaluated on COVID-19 patients and validated with a Hemathix scanning capillary viscometer, the Walburn–Schneck model was found to underestimate WBV particularly at a low-shear rate; however, the model retained a moderate-to-high correlation between WBV and eBV [21]. Secondly, as data were collected over the course of several months, viral variants, vaccination status, and hospital protocols may have varied by admission date. This variable was addressed in subgroup analysis. Thirdly, we were unable to perform a direct comparison with other established prognostic tools such as the SOFA score or APACHE II score due to insufficient data. However, we attempted to supplement our analysis by including the PaO_2_/FiO_2_ ratio and other inflammatory markers to evaluate the additional prognostic value provided by eBV. Although the results were not statistically significant likely due to limited statistical power in this subgroup, participants with higher eHSBV still exhibited a trend towards lower odds for respiratory organ support-free days even after adjustment for PaO_2_/FiO_2_ ratio. Finally, as an observational retrospective trial, there may be unidentified confounders that potentially impact our associations. Although multiple possible covariates were considered, future prospective studies will be needed to confirm the findings.

Despite these limitations, our findings are highly relevant clinically as they demonstrate the ability of eHSBV and eLSBV to predict non-fatal COVID-19 outcomes early in the disease course. The associations observed in our investigation were consistent across a range of covariates, disease severities and provide a method of risk stratification after adjustment for age, sex and cardiometabolic comorbidities; inflammatory biomarkers including WBC, CRP, D-dimer, Furthermore, as variables required for the calculation of eBV utilizing the Walburn–Schneck model are easily obtained from routinely drawn laboratory values (hematocrit, albumin, total protein), our study demonstrates a proof of concept of a measure accessible to practitioners. Finally, given the associations between blood hyperviscosity and the reduced likelihood of being respiratory organ support-free at 21 days, future COVID-19 therapeutics may be targeted at modalities that reduce blood viscosity.

## Data Availability

Formal requests to access the dataset need to be sent to the COVID-19 Committee, Icahn School of Medicine.

## Abbreviations

aOR: Adjusted odds ratio
CRP: C-reactive protein
cP: Centipoise
CI: Confidence Interval
COVID-19: Coronavirus disease 2019
WBV: Whole blood viscosity
eWBV: Estimated whole blood viscosity
eHSBV: Estimated high-shear blood viscosity
eLSBV: Estimated low-shear blood viscosity
ICU: Intensive Care Unit
MSHS: Mount Sinai Health System
WBC: White blood cell count
